# A paradigm shift in DENV-4 clinical presentation: A viewpoint on pulmonary inflammatory symptoms from the 2023 Lincang outbreak

**DOI:** 10.1371/journal.pntd.0013679

**Published:** 2025-11-07

**Authors:** Bo Zhang, Fuying Guo, Peng Wang, Xiangyu Yan, Wei Li, Huakun Xu, Shuo Kou, Changxian Lu, Ling Zhang, Tiejun Shui

**Affiliations:** 1 School of Environmental Science and Engineering, Hainan University, Haikou, China; 2 The People’s Hospital of Lincang, Lincang, Yunnan, China; 3 Department of Epidemiology and Biostatistics, Memorial Sloan Kettering Cancer Center, New York, New York, United States of America; 4 School of Disaster and Emergency Medicine, Tianjin University, Tianjin, China; 5 Yunnan Provincial Hospital of Traditional Chinese Medicine, Kunming, China; 6 Xishuangbanna Dai Autonomous Prefecture People’s Hospital, Jinghong, China; 7 Yunnan Center for Disease Control and Prevention, Kunming, China; University of Dhaka, BANGLADESH

## Abstract

**Background:**

During the 2023 outbreak of dengue virus serotype 4 (DENV-4) in Lincang, China, we noted prominent lower-respiratory involvement not typically emphasized in dengue descriptions.

**Methods:**

We retrospectively analyzed 147 hospitalized, RT-PCR-confirmed DENV-4 cases. Respiratory assessment followed a tiered protocol: daily symptom screening and pulse oximetry for all patients; chest radiography and point-of-care lung ultrasound as indicated; and chest CT for severe or atypical presentations. Symptom structure was evaluated using EBICglasso network analysis. Predictors of severe dengue were examined with logistic regression.

**Results:**

Pulmonary symptoms occurred in 31.3% (46/147), most frequently lower respiratory infection (28.6%), cough (25.9%), and chest distress (15.0%). Symptoms resolved quickly with supportive care (median 7 days, IQR 6–9), with no residual pulmonary sequelae at discharge. Network analysis identified a cohesive respiratory cluster with regularized partial correlations of 0.19–0.23, indicating robust conditional associations among pulmonary symptoms within the overall clinical spectrum. In regression models, thrombocytopenia (Adjusted Odds Ratio, AOR 3.06, 95% CI 1.05–9.03) and hepatic hypofunction (AOR 3.82, 95% CI 1.00–13.61) were independently associated with severe dengue; coexisting lower respiratory infection was associated with severity in univariate analysis (Odds Ratio, OR 2.91, 95% CI 1.05–8.07). Sex-specific patterns emerged: females more often had weakness, leukopenia, headache, and hypokalemia, whereas males had higher rates of hyperuricemia.

**Conclusions:**

In this genotype I DENV-4 cohort, lower-respiratory manifestations were common, formed a coherent symptom network, and showed signals of association with severity. These findings support routine respiratory screening, pulse oximetry, and stepwise imaging in dengue care pathways, with validation needed in multicenter studies.

## 1. Introduction

The dengue virus (DENV), a flavivirus with four distinct serotypes, poses a significant and growing threat to global public health, with an estimated 390 million infections occurring annually [1]. The clinical spectrum ranges from asymptomatic infection to life-threatening dengue hemorrhagic fever and dengue shock syndrome. Historically, the primary symptoms have been fever, bleeding, and fluid leakage from blood vessels.

However, emerging evidence challenges this traditional understanding. Recent outbreaks demonstrate that dengue’s clinical spectrum extends well beyond classical hepatic and hematological manifestations. Systematic reviews of clinical and radiological data reveal that pulmonary involvement, though historically underappreciated, represents a significant component of dengue pathophysiology [2].

Pulmonary involvement in dengue has been increasingly documented across diverse geographic regions and serotypes. A systematic analysis of recent literature reveals consistent patterns: Rodrigues and colleagues (2014) documented pleural effusion in 55.2% and ground-glass opacities in 27.6% of 2,020 Brazilian patients with predominantly DENV-1 and DENV-2 infections [3]. Similarly, Almeida and colleagues’ (2017) systematic review spanning 56 years established a hierarchy of pulmonary manifestations: pleural effusion (17%–100%), noncardiogenic pulmonary edema (2%–29%), and diffuse alveolar hemorrhage (<1%) [2]. More recent hospital-based cohorts provide clinical context: Gupta and colleagues (2020) reported 56% pulmonary complications in an Indian ICU setting with 22% developing acute respiratory distress syndrome [4], while Keshav and colleagues (2024) documented lower prevalence (10.2%) but higher mortality (23%) among those with pulmonary involvement [5]. Notably, these studies predominantly examined DENV-1, DENV-2, and DENV-3, highlighting a critical gap in DENV-4-specific respiratory manifestations that our study addresses.

These studies reveal a critical knowledge gap: although pulmonary manifestations have been documented in DENV-1, DENV-2, and DENV-3 infections, the literature lacks systematic characterization of DENV-4-associated respiratory complications, especially in primary outbreaks in populations with no prior exposure. This gap is particularly important given the different immunopathological profiles and clinical severity patterns observed among the serotypes. These findings suggest that DENV may have unique pulmonary tropism characteristics that require further investigation.

**Fig 1:**
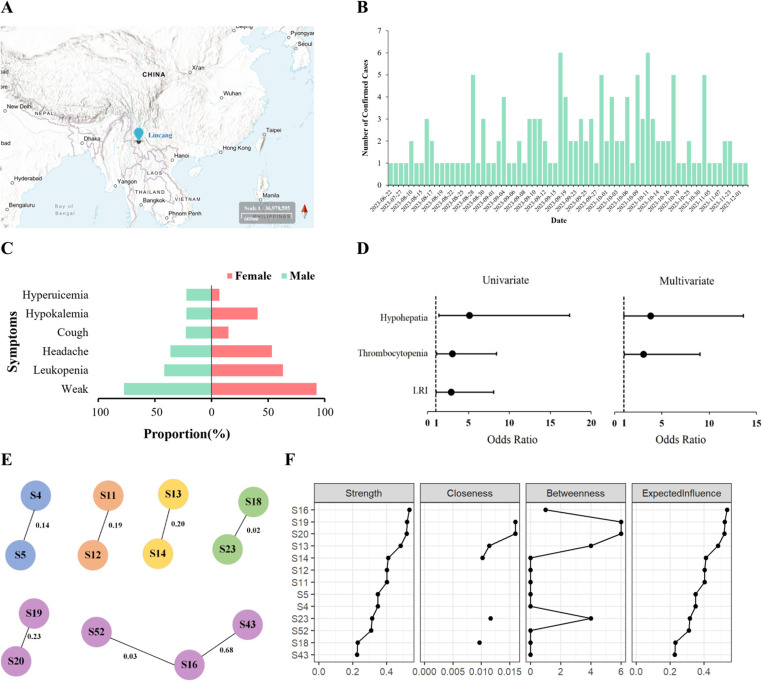
Epidemiological, temporal, clinical, and indicator symptoms for severe dengue of the 2023 dengue fever outbreak in Lincang, China. **A**, Geographic location of Lincang. Base map: U.S. Geological Survey (public domain), usgs.gov. Map compilation and annotations by the authors. **B**, Temporal trend of the dengue fever outbreak. **C**, Sex-specific differences in clinical symptoms. **D**, Indicator symptoms for severe dengue fever. **E**, Visualization of the symptom network. This figure presents a graphical representation of the symptom network derived from dengue fever cases. Only statistically significant associations between symptoms (*p* < 0.05) are included, with nodes representing symptoms and edges indicating their correlations. The weight of the edges reflects the strength of the associations. Refer to Table 1 in S1 Table for the symptom codes. **F**, Centrality measures of symptoms in the network.

In 2023, Lincang City in Yunnan Province, China, experienced a notable dengue fever outbreak caused by Dengue Virus Serotype 4 (DENV-4) (Fig 1A). Yunnan has historically been recognized as a dengue hotspot in mainland China, partly due to its favorable climatic conditions that support the breeding of *Aedes* mosquitoes [1]. While previous studies have primarily emphasized the hemorrhagic manifestations and hepatic or hematological complications of dengue, the 2023 outbreak emphasized the importance of pulmonary symptoms in disease progression. This viewpoint synthesizes findings from 147 confirmed cases in Lincang and emphasizes why pulmonary symptoms warrant more clinical and research attention. By drawing upon newly published studies and contextualizing the outbreak within the evolving clinical profile of dengue, we aim to provide insights that are relevant to clinicians, public health professionals, and researchers of neglected tropical diseases.

## 2. Ethics statement

Formal written consent was obtained from all participants and their parents/guardians prior to their involvement in the study. The consent process ensured that both the participants and their parents/guardians were fully informed about the purpose, procedures, and potential risks of the study. The ethical approval was provided by the Yunnan Center for Disease Control and Prevention (No.QR-KJB-2021-003).

## 3. Laboratory confirmation and serotype assignment

The virological diagnosis was conducted in accordance with the National Laboratory Testing Scheme for Dengue, as stipulated by the China CDC (2023) [6,7]. Acute-phase sera (≤5 days post-onset) underwent NS1 antigen capture immunochromatography (Wantai BioPharm) and multiplex real-time RT-PCR targeting the C-prM junction using serotype-specific probes (Shengxiang Biotechnology, CFDA No. SXDD-04). All RT-PCR-positive samples (*n* = 147) yielded DENV-4 amplification with Ct values ranging from 18.3 to 36.7 (median 28.4).

In order to satisfy the national demand for molecular epidemiological surveillance during outbreaks, E-gene sequencing (1,485 bp) was performed on all specimens. Phylogenetic analysis employing the MEGA 11.0 software program confirmed that all sequences fell within the DENV-4 genotype I cluster.

For convalescent sera with a duration of infection greater than five days or for cases in which the results of the reverse transcription-polymerase chain reaction (RT-PCR) were inconclusive, a paired immunoglobulin M (IgM)/IgG enzyme-linked immunosorbent assay (ELISA) was performed using the Panbio kit. The presence of seroconversion or a fourfold increase in titer was indicative of an acute infection. The quality assurance program encompassed the validation of the specimens by a provincial reference laboratory and the utilization of national standard positive/negative control panels. The analysis was conducted exclusively on cases that satisfied the criteria for dual-assay confirmation (S1 Text).

## 4. Epidemiological context

Between August and October 2023, 147 cases of dengue fever (all confirmed to be DENV-4 infections) were hospitalized at the People’s Hospital of Lincang, a city located near the Myanmar border (Fig 1B). Epidemiological investigations indicated that 51.7% (76/147) of the patients were male, with a median age of 43 years (interquartile range [IQR]: 33–58). Approximately 76.2% of the cases originated from Gengma County, a region frequently noted for its dense *Aedes* mosquito population [8]. These demographic parameters are consistent with prior dengue incidence trends in Yunnan, although the 2023 outbreak demonstrated unique clinical features that differentiate it from previous episodes in the same area [9].

Key contributing factors to the increasing dengue incidence in these regions include rising temperatures, climatic changes, increased mobility, and cross-border movement [10]. Indeed, the spillover of dengue outbreaks across neighboring countries has been observed, facilitated by porous borders and fluctuating mosquito vector burdens. This underscores the complexity of controlling dengue, as any local outbreak could potentially assume a regional dimension. Furthermore, the COVID-19 pandemic has likely exacerbated diagnostic challenges, as limited healthcare resources and overlapping symptoms may have contributed to delayed dengue detection in some patients [11].

## 5. Clinical presentation and methodological overview

All 147 hospitalized cases underwent standardized clinical and virological evaluation. Comorbidities were classified using to ICD-10 codes, and disease severity was classified per established guidelines (Table A in S1 Table). Notably, the median hospitalization duration was seven days (IQR: 6–9), and the median maximum body temperature reached 39.0°C (IQR: 38.1–39.3°C) (Table B in S1 Table). These values align with typical dengue presentations seen globally, although the range of “febrile phases” extends considerably among patients [12].

Pulmonary assessment was conducted in accordance with the 2023 National Dengue Prevention and Control Protocol, employing a tiered approach. At admission and subsequently on a daily basis, all patients underwent a standardized respiratory screening procedure. This procedure included a review of symptoms such as cough, dyspnea, chest pain, and sputum. In addition, a physical examination with auscultation was performed, and the respiratory rate and room-air SpO₂ were recorded. When receiving oxygen, the patient’s position and the FiO₂ were documented. The diagnostic approach was meticulous and judicious, entailing a sequential process of evaluation. Initially, chest X-rays were obtained to ascertain the presence of respiratory symptoms, abnormal lung findings, or hypoxemia. Subsequently, when feasible, handheld point-of-care lung ultrasonography was employed to assess interstitial changes and pleural effusions. Finally, chest CTs were reserved for cases that met predefined criteria, such as severe, non-resolving, or atypical cases, where the results were anticipated to lead to a modification in management.

Patients were monitored daily for symptom evolution. Resolution was defined as the first 24-hour period free of chest distress, dyspnea, cough and expectoration without the need for supplemental oxygen or bronchodilators. Time-to-resolution (TTR) was calculated from hospital admission to symptom clearance.

We employed EBICglasso network analysis to identify symptom relationships. This method uses regularized partial correlations, which differ from simple correlations by controlling for all other symptoms simultaneously [13]. For example, the correlation between chest distress and dyspnea (edge weight = 0.23) represents their direct association after removing the influence of all other symptoms. The LASSO penalty eliminates spurious connections, ensuring only robust associations remain. Consequently, the emergence of the pulmonary cluster with edge weights ranging from 0.19 to 0.23, although seemingly modest, signifies the presence of robust conditional dependencies that have endured rigorous statistical filtration. From a clinical perspective, this grouping of symptoms indicates that they represent a cohesive syndrome rather than distinct manifestations. This finding is significant because it has substantial implications for screening methodologies and management strategies (see Fig 1E and 1F).

## 6. Sex-specific patterns

Sex-specific differences led to interesting observations. Females more frequently reported weakness (93.0%), leukopenia (63.4%), headaches (53.5%), and hypokalemia (40.8%), while males showed higher tendencies for hyperuricemia (22.4%) and splenomegaly (11.8%). The incidence of splenomegaly in dengue, though not the most classical finding, can occur and should not be disregarded when assessing patient outcomes (Fig 1C, Table C in S1 Table).

An accumulating body of evidence indicates that sex differences in dengue reflect intertwined biological and socio-behavioral processes rather than a single causal mechanism. On the biological axis, estrogens modulate endothelial tone and barrier integrity through nitric oxide-dependent and receptor-mediated signaling, plausibly influencing the propensity to plasma leakage that underlies severe disease, while broader sexual dimorphism in immune responses can shape viremia kinetics, inflammatory profiles, and downstream clinical manifestations [14].

Experimental and translational studies demonstrate that estrogens enhance endothelial resilience and reduce paracellular permeability, with potential implications for pulmonary and serosal fluid shifts during critical-phase dengue, whereas females generally mount more robust innate and humoral responses, which may alter both susceptibility and phenotype expression across the disease course.

It is hypothesized that socio-behavioral determinants likely operate in parallel with biological mechanisms, contributing to sex-differential dengue outcomes. It has been demonstrated that men may incur greater vector exposure through occupational and outdoor activities. In contrast, women in some settings face delayed access to formal care or preferential reliance on traditional providers. These dynamics have the potential to generate surveillance artifacts and presentation delays, which can lead to the inflation of apparent clinical disparities that are not necessarily indicative of biological differences.

A pre-specified framework will differentiate these pathways by: The integration of sex- and stage-specific factors (menstrual/menopausal status, pregnancy, exogenous hormone use) with immune and endothelial biomarkers is a crucial aspect of the study. The quantification of exposure and access variables, including but not limited to occupation, outdoor time, protective practices, household vector control, time-to-presentation, and decision-making norms, is essential for a comprehensive understanding of the subject matter. The application of sex-stratified models, incorporating interaction terms and causal mediation, aims to partition total effects into biological and socio-behavioral components. This approach is supplemented by sensitivity analyses for unmeasured confounding and differential misclassification in symptom reporting or care-seeking.

From a clinical perspective, a dual-action strategy is recommended: first, implement sex-aware respiratory risk appraisal and conservative fluid stewardship for patients with lower-respiratory symptoms or hypoxemia (reflecting potential biological risk); second, concurrently strengthen outreach and triage access to reduce care-seeking delays and exposure-driven risk. To address this gap, routine equity monitoring should be implemented to detect and remediate sex-differential gaps in processes and outcomes.

## 7. Pulmonary symptoms: An emerging concern

Pulmonary symptoms were present in 46 patients (31.3%). The most frequent complaint was cough (38/147, 25.9%), followed by dyspnea (29/147, 19.7%), and chest distress (22/147, 15.0%).

The respiratory symptoms resolved rapidly under the administration of supportive care. The median TTR was found to be 4 days (IQR 3–6) overall and 6 days (IQR 5–8) in severe cases. A total of 46 symptomatic patients demonstrated complete resolution by day 14 of follow-up, with no evidence of residual pulmonary sequelae.

The prevalence of lower respiratory symptoms in the Lincang outbreak appears to be higher than that observed in general ward-based dengue cohorts, but lower than that seen in severe/critical care strata. In Brazil, Rodrigues and colleagues (2014) evaluated a highly selected subset of 29 dengue patients who underwent chest CT because of respiratory complaints and reported cough in 27.6% and dyspnea in 37.9% within that CT subset; only 1.43% of the broader 2,020 case cohort underwent CT, so these figures are not population prevalences [2]. In India, Gupta and colleagues (2020) found that among hospitalized dengue patients with pulmonary complications, dyspnea and cough occurred in 92.8% and 78.6%, respectively, compared with 9.0% and 54.5% in those without pulmonary complications [4]. In a larger Indian inpatient cohort, Keshav and colleagues (2024) observed overall cough and dyspnea rates of 16.1% and 5.5% across 255 patients, rising to 26.9% and 30.8% among those with lung involvement [5]. In this context, the DENV-4 figures in Lincang (25.9% for cough and 28.6% for LRI) exceed the routine ward level averages and are comparable to selected imaging-triggered series. However, they remain well below the frequencies observed in severe disease with pulmonary complications [2,4,5].

The network analysis revealed that pulmonary symptoms form a distinct cluster with edge weights (regularized partial correlations) of 0.19–0.23. These values, which represent conditional associations subsequent to controlling for all other symptoms, indicate robust direct relationships within this syndromic complex. Despite the presence of moderate individual edge weights, the emergence of the cluster indicates a unified pathophysiological process that possesses substantial prognostic value. Keshav and colleagues reported an odds ratio of 6.7 (95% CI 3.2–13.9) for “positive lung imaging to death” [5]. Collectively, the data delineate a step-up pattern from symptoms to imaging positivity to fatality, thereby emphasizing the feasibility of early symptom-based triage.

Our serotype-specific data and symptom-network analysis complement radiological and autopsy literature, indicating that “pulmonary linkage” is an evolving component of the dengue clinical spectrum rather than a mere complication. From a pathophysiological standpoint, pulmonary involvement in dengue likely arises from a synergistic interplay between the virus’s direct effect on capillary and endothelial function, immune-mediated inflammatory cascades, and possible secondary bacterial or viral infections [15]. When alveolar-capillary integrity is compromised, fluid leakage into pulmonary interstitial can precipitate acute respiratory distress. This phenomenon underscores the importance of early intervention, including fluid management, oxygen supplementation, and strict monitoring of respiratory parameters, particularly in settings where dengue presentations have diversified beyond the classical febrile-hemorrhagic spectrum.

## 8. Predictors of severe disease

Univariate and multivariable logistic regression analyses pinpointed thrombocytopenia and hypoplasia (diminished liver function) as significantly associated with severe dengue fever (Fig 1D, Table D in S1 Table). Thrombocytopenia (OR = 3.06, 95% CI: 1.05–9.03) emerged as a well-known and robust predictor, aligning with longstanding research on platelet count as a core marker of disease severity in dengue [16]. Hyperpathia (OR = 3.82, 95% CI: 1.00–13.61) likewise flagged disease severity, consistent with previous outbreaks where hepatic involvement was a key indicator of poor outcomes [17]. Notably, lower respiratory infection coexisting with dengue had an OR of 2.91 (95% CI: 1.05–8.07) in univariate analysis, further highlighting the significance of respiratory system stress in severe clinical phenotypes.

Potential interactions between systemic and pulmonary manifestations remain incompletely understood. For example, the synergy between hepatic dysfunction and inflammatory cascades could predispose alveolar and bronchial tissues to exudative processes. Although the current study’s sample size was modest (*n* = 147), the strength of associations between pulmonary symptoms and severity underscores the need for further large-scale research. It also signals that comprehensive assessment of respiratory parameters (e.g., imaging studies, arterial blood gases) might become more standard in resource-limited settings wherein dengue remains endemic.

## 9. Implications for clinical management and research

Lower-respiratory manifestations of dengue, including chest discomfort, dyspnea, cough, and sputum production, should be explicitly integrated into routine assessment and triage within existing dengue pathways. These features serve as clinically accessible indicators of systemic inflammation and plasma leakage, which can be detected using scalable, low-cost tools such as standardized symptom inquiry and pulse oximetry. Consequently, they are well-suited for implementation in resource-limited settings.

### 9.1 Clinical management

The process of screening and escalation is a critical component of the larger framework of care. It is imperative to incorporate a respiratory-focused screen at the time of admission and during daily medical assessments. This screen should encompass a structured assessment of symptoms, in conjunction with the measurement of respiratory rate and the SpO_2_ levels present in the room air. Conversely, positive findings should initiate an escalation pathway that calibrates monitoring frequency, fluid stewardship, and oxygen supplementation. This pathway operationalizes the World Health Organization’s warning signs by converting respiratory complaints and hypoxemia into actionable triage triggers.

The rapid symptom clearance (median 4 days, 100% by day 14) indicates that pulmonary manifestations in DENV-4 are largely self-limiting when managed according to current guideline-based supportive protocols. This favorable prognosis contrasts with the protracted recovery seen in some viral pneumonias and supports the hypothesis that dengue-related pulmonary symptoms primarily reflect transient capillary leak rather than direct alveolar damage.

### 9.2 Diagnostic approach

Universal pulse oximetry is the primary imaging modality when symptoms, abnormal examination findings, or hypoxemia are present. Chest radiography is the imaging modality of choice in these cases.

The utilization of selective handheld point-of-care ultrasound (POCUS) is recommended, when available, to assess interstitial involvement and pleural effusions. This approach aims to refine bedside decisions regarding fluids and oxygen therapy.

The third component of the diagnostic evaluation includes targeted microbiologic testing. This testing should include basic inflammatory markers, blood cultures for suspected sepsis, sputum studies for productive cough or consolidation, and rapid antigen or PCR assays for prevalent respiratory viruses when feasible.

### 9.3 Therapeutic principles

It is imperative to prioritize conservative fluid administration, accompanied by meticulous surveillance for potential plasma leakage. Early oxygen support is crucial for addressing hypoxemia, and judicious use of antibiotics is paramount—reserving empiric therapy for cases with convergent clinical, radiographic, and laboratory evidence of bacterial pneumonia.

### 9.4 Recommendation for standardized assessment protocol

Initial Assessment (Emergency Department/Admission):

(1) Respiratory symptom screen: cough, dyspnea, chest pain, sputum production;
(2) Vital signs including respiratory rate and room-air SpO_2_;
(3) Risk stratification:
  - Low risk (SpO_2_ ≥95%, no distress): Standard dengue monitoring.
  - Intermediate risk (SpO_2_ 90%–94% or mild distress): Initiate oxygen, obtain chest X-ray, consider POCUS.
  - High risk (SpO_2_ <90% or severe distress): Immediate oxygen, chest imaging, ICU evaluation.

Daily Monitoring:

(1) Symptom evolution tracking using standardized checklist.
(2) SpO_2_ measurement every 8 hours (every 4 hours if intermediate/high risk).
(3) Escalation triggers clearly defined and nurse-initiated.

### 9.5 Resource-stratified implementation

In light of the acknowledged heterogeneity in healthcare capacities across endemic settings, the implementation of the proposed respiratory assessment framework should be explicitly resource-stratified. In settings with limited resources, a pragmatic approach centered on standardized, paper-based symptom checklists, routine pulse oximetry at admission and daily thereafter, and predefined clinical triggers for escalation can ensure timely identification of patients at risk of respiratory compromise. Basic-resource settings should augment this foundation with electronic tracking systems where feasible, selective chest radiography guided by clinical criteria, and limited POCUS performed by clinicians with focused, short-course training to detect interstitial syndromes (e.g., B-lines) and pleural effusions. In standard-resource settings, comprehensive imaging (including chest CT when indicated), arterial blood gas analysis, and multidisciplinary consultation (infectious diseases, pulmonology, and critical care) can be integrated to refine diagnosis, monitor disease trajectory, and guide escalation, including ICU transfer.

Across all tiers, the framework emphasizes scalability, safety, and fidelity to core decision nodes. It is imperative to preserve thresholds for oxygen supplementation (e.g., SpO_2_ <94% with symptoms or <90% irrespective of symptoms), standardized reassessment intervals, and nurse-initiated escalation pathways. However, diagnostic intensity and monitoring frequency must be calibrated to local capacity. This stratified approach facilitates consistent risk detection and clinical decision-making without overburdening constrained systems, while enabling higher-capacity centers to leverage advanced diagnostics for precision management and outcomes optimization.

### 9.6 Sex-specific and equity considerations

Incorporate sex-specific factors into triage protocols, acknowledging differential exposure profiles and care-seeking behaviors. Implement equity monitoring through sex-stratified dashboards tracking time-to-triage, oxygen initiation, and clinical outcomes. Pregnancy requires enhanced maternal-fetal monitoring with tailored hemodynamic management according to obstetric guidelines.

### 9.7 Research priorities

Research priorities center on establishing multi-center prospective cohorts stratified by sex and physiological stage, underpinned by harmonized biobanking to enable longitudinal multi-omics. These cohorts should integrate comprehensive measurement of biological mechanisms (sex hormones, immune/endothelial activation markers, viral kinetics, genomic features), behavioral determinants (occupation-related exposure, care-seeking patterns, comorbidities) using standardized instruments, vector surveillance (household and community entomology linked to environmental mapping), and standardized clinical phenotyping (WHO warning signs, respiratory manifestations, oxygenation indices, sex-specific outcomes via unified case report forms). The analytical rigor of the study will be ensured through the use of pre-specified causal diagrams, multilevel models with interaction terms, symptom-network analyses, and formal mediation to partition sex effects. Quality assurance will include preregistration, centralized training, batch-controlled assays, shared data dictionaries, and principled handling of missing data. Ethical safeguards will encompass tiered consent for genetic analyses, inclusion of pregnant participants with appropriate oversight, and sustained community engagement.

## 10. Conclusion

This study fundamentally challenges our understanding of DENV-4 clinical presentation. The high prevalence of pulmonary manifestations (43.5%), their central role in the symptom network, and strong association with disease severity demand immediate integration into clinical protocols. While our findings require validation in larger, multi-center studies, the evidence compels a paradigm shift: respiratory assessment must become a cornerstone of dengue management, not an afterthought. As climate change expands dengue’s geographic reach, recognizing and managing these pulmonary manifestations will be critical for reducing morbidity and mortality across endemic regions.

However, these conclusions are tempered by important limitations. The analysis derives from a modest sample within a single outbreak, and imaging and microbiologic testing were symptom- and resource-triggered rather than universal. This approach potentially enriches the sample for severe phenotypes and introduces selection bias and misclassification. Consequently, the findings warrant cautious interpretation and prospective validation. The following areas have been identified as priorities: multicenter studies to confirm associations, elucidate the pathophysiological links between systemic inflammation, plasma leakage, and respiratory phenotypes, and refine therapeutic approaches that balance conservative fluid strategies with timely oxygen support and judicious antimicrobial use. Proactive research, in conjunction with integrated, respiratory-focused monitoring, will be essential to mitigate complications and reduce dengue-related mortality as clinical presentations continue to evolve.

## Supporting information

S1 Text Definition of dengue and severe dengue. (DOCX)

S1 Table **Table A.** Symptoms and ICD-10 codes. **Table B.** Base characteristics of dengue patients, Yunnan Lincang, 2023. **Table C.** Clinical symptoms of dengue patients, Yunnan Lincang, 2023. **Table D.** The univariate and multivariate analysis of severe dengue infection.
(DOCX)
