# Supplementary material for: A paradigm shift in DENV-4 clinical presentation: A viewpoint on pulmonary inflammatory symptoms from the 2023 Lincang outbreak
Source: PLoS Negl Trop Dis. 2025 Nov 7;19(11):e0013679. doi: 10.1371/journal.pntd.0013679 (PMC12594406; doi:10.1371/journal.pntd.0013679)
Supplement: S1 Text — (DOCX) [file pntd.0013679.s001.docx]

**Definition of Dengue Fever and Severe Dengue**

**Dengue Fever**

According to the Dengue Fever Diagnosis and Treatment Guidelines (2024 Edition), a clinical diagnosis of dengue fever is confirmed if one of the following criteria is met:

1. Positive detection of dengue virus nucleic acid;
2. Isolation and culture of the dengue virus;
3. Serological testing shows seroconversion of dengue virus IgG antibodies or a fourfold or greater increase in antibody titers during the recovery phase compared to the acute phase.

**Severe Dengue**

A diagnosis of severe dengue is made if one or more of the following conditions are present:

1. Severe bleeding: Such as subcutaneous hematomas, hematemesis, melena, vaginal bleeding, gross hematuria, intracranial hemorrhage, etc.
2. Shock: Characterized by tachycardia, cold and clammy extremities, capillary refill time greater than 3 seconds, weak or undetectable pulse, reduced pulse pressure, or blood pressure below 90/60 mmHg, or a drop in blood pressure of more than 20% from baseline.
3. Severe organ impairment, meeting one or more of the following criteria:

(1) Liver damage: Serum ALT and/or AST > 1000 IU/L or total bilirubin > 85.5 μmol/L;

(2) Kidney damage: Serum creatinine > 176.8 μmol/L or above the upper limit of normal and exceeding baseline values by 2 times, or urine output less than 0.5 ml/(kg·h) for at least 24 hours;

(3) Cardiac involvement: Heart failure, cardiogenic shock, malignant arrhythmias, etc.;

(4) Neurological involvement: Encephalopathy, encephalitis, myelitis, Guillain-Barré syndrome, etc.;

(5) Others: Rhabdomyolysis, acute pancreatitis.
