# Supplementary material for: A paradigm shift in DENV-4 clinical presentation: A viewpoint on pulmonary inflammatory symptoms from the 2023 Lincang outbreak
Source: PLoS Negl Trop Dis. 2025 Nov 7;19(11):e0013679. doi: 10.1371/journal.pntd.0013679 (PMC12594406; doi:10.1371/journal.pntd.0013679)
Supplement: S1 Table — Table A. Symptoms and ICD-10 codes. Table B. Base characteristics of dengue patients, Yunnan Lincang, 2023. Table C. Clinical symptoms of dengue patients, Yunnan Lincang, 2023. Table D. The univariate and multivariate analysis of severe dengue infection. (DOCX) [file pntd.0013679.s002.docx]

**Table A Symptoms and ICD-10 codes**

| No. | Symptom | ICD-10 |
| --- | --- | --- |
| S1 | Fever | R50 |
| S2 | Chilly | R68.84 |
| S3 | Chill | R68.83 |
| S4 | Weak | R53.1 |
| S5 | Poor appetite | R63.0 |
| S6 | Stiff | M79.1 |
| S7 | Nausea | R11.0 |
| S8 | Emesis | R11.2 |
| S9 | Diarrhea | R19.7 |
| S10 | Stomachache | R10.9 |
| S11 | Rash | R21 |
| S12 | Skin itch | L29.9 |
| S13 | Cough | R05 |
| S14 | Expectoration | R09.3 |
| S15 | Throat pain | R07.0 |
| S16 | Orbital pain | H57.1 |
| S17 | Headache | R51 |
| S18 | Swirl | R42 |
| S19 | Chest distress | R07.9 |
| S20 | Anhelation | R06.0 |
| S21 | Breathing difficulties | R06.2 |
| S22 | Flustered | R00.0 |
| S23 | Palpitation | R00.2 |
| S24 | Epistaxis | R04.0 |
| S25 | Bleeding gums | K05.2 |
| S26 | Pharyngohemia | J02.9 |
| S27 | Palpebral conjunctival hyperemia | H11.3 |
| S28 | Antiadoncus | J35.0 |
| S29 | Face red | R23.0 |
| S30 | Scheming damage | I51.9 |
| S31 | Liver damage | K76.9 |
| S32 | Leukopenia | D72.8 |
| S33 | Thrombocytopenia | D69.6 |
| S34 | Mild anemia | D64.0 |
| S35 | Moderate anemia | D64.1 |
| S36 | Severe anemia | D64.2 |
| S37 | Hyperuricemia | E79.0 |
| S38 | Electrolyte metabolism disorder | E87.9 |
| S39 | Hypokalemia | E87.6 |
| S40 | Hyponatremia | E87.1 |
| S41 | Hypocalcemia | E83.5 |
| S42 | Hypoproteinemia | E46 |
| S43 | Hyperlipidaemia | E78.5 |
| S44 | Splenauxe | R16.0 |
| S45 | Cyst of kidney | Q61.9 |
| S46 | Acute renal insufficiency | N17.9 |
| S47 | Acute respiratory distress syndrome | J80 |
| S48 | Upper respiratory infection (URI) | J06.9 |
| S49 | Lower respiratory infection (LRI) | J22 |
| S50 | Lacunar infarction | I63.9 |
| S51 | Type 2 diabetes | E11 |
| S52 | Primary hypertension | I10 |
| S53 | Secondary hypertension | I11 |
| S54 | Tertiary hypertension | I12 |
| S55 | Hypohepatia | K76.9 |
| S56 | Gastrointestinal dysfunction | K59.9 |
| S57 | Pleurisy | J84.1 |
| S58 | Rickettsial infection | A77.9 |
| S59 | Lung Nodule | R91.1 |

Table B. Base characteristics of dengue patients, Yunnan Lincang, 2023

| **Characteristic** | **Total** | **Male** | **Female** | **p** |
| --- | --- | --- | --- | --- |
| Total | 147 | 76 | 71 |  |
| Age, Median (IQR) | 43.0(33.0-57.5) | 43.5(33.0-59.3) | 43.0(33.5-54.5) | 0.63 |
| Age group |  |  |  | 0.68 |
| ≤10 | 9(6.1) | 6(7.9) | 3(4.2) |  |
| 11~20 | 10(6.8) | 5(6.6) | 5(7.0) |  |
| 21~30 | 10(6.8) | 6(7.9) | 4(5.6) |  |
| 31~40 | 37(25.2) | 15(19.7) | 22(31.0) |  |
| 41~50 | 32(21.8) | 17(22.4) | 15(21.1) |  |
| 51~60 | 17(11.6) | 9(11.8) | 8(11.3) |  |
| 61~70 | 20(13.6) | 13(17.1) | 7(9.9) |  |
| 71~ | 12(8.2) | 5(6.6) | 7(9.9) |  |
| Ethnicity |  |  |  | 0.90 |
| Han | 110(74.8) | 58(76.3) | 52(73.2) |  |
| Dai | 23(15.6) | 11(14.5) | 12(16.9) |  |
| Other | 14(9.6) | 7(9.2) | 7(9.1) |  |
| Geographic location |  |  |  | 1.00 |
| Gengma | 112(76.2) | 58(76.3) | 54(76.1) |  |
| Other counties | 35(23.8) | 18(23.7) | 17(23.9) |  |
| Severity of dengue fever |  |  |  | 0.92 |
| Classic | 129(87.8) | 66(86.8) | 63(88.7) |  |
| Severe | 18(12.2) | 10(13.1) | 8(11.3) |  |
| Days of hospitalization, Median (IQR) | 7(6-9) | 8(6-10) | 7(6-9) | 0.20 |
| Maximal body temperature, Median (IQR) | 39.0(38.1-39.3) | 39.0(38.3-39.4) | 38.9(38.0-39.2) | 0.11 |
| Comorbidities |  |  |  |  |
| Type 2 diabetes | 12(8.2) | 3(3.9) | 9(12.7) | 0.08 |
| Primary hypertension | 2(1.4) | 1(1.3) | 1(1.4) | 1.00 |
| Secondary hypertension | 8(5.4) | 3(3.9) | 5(7.0) | 0.47 |
| Tertiary hypertension | 6(4.1) | 3(3.9) | 3(4.2) | 1.00 |
| Lacunar infarction | 6(4.1) | 4(5.3) | 2(2.8) | 0.70 |
| Cyst of kidney | 31(21.1) | 19(25.0) | 12(16.9) | 0.30 |
| Splenauxe | 10(6.8) | 9(11.8) | 1(1.4) | 0.01 |
| Hypocalcemia | 8(5.4) | 4(5.3) | 4(5.6) | 1.00 |
| Severe anemia | 5(3.4) | 3(3.9) | 2(2.8) | 1.00 |

Table C. Clinical symptoms of dengue patients, Yunnan Lincang, 2023

| **Characteristic** | **Total** | **Male** | **Female** | **p** |
| --- | --- | --- | --- | --- |
| Total | 147 | 76 | 71 |  |
| Clinical Symptoms |  |  |  |  |
| Fever | 140(95.2) | 73(96.1) | 67(94.4) | 0.70 |
| Weakness | 125(85.0) | 59(77.6) | 66(93.0) | 0.01 |
| Poor appetite | 108(73.5) | 54(71.1) | 54(76.1) | 0.59 |
| Stiff | 106(72.1) | 53(69.7) | 53(74.6) | 0.58 |
| Chilly | 91(61.9) | 46(60.5) | 45(63.4) | 0.44 |
| Leukopenia | 77(52.4) | 32(42.1) | 45(63.4) | 0.01 |
| Nausea | 66(44.9) | 31(40.8) | 35(49.3) | 0.33 |
| Headache | 66(44.9) | 28(36.8) | 38(53.5) | 0.04 |
| Liver damage | 51(34.7) | 31(40.8) | 20(28.2) | 0.13 |
| Lung Nodule | 48(32.7) | 27(35.5) | 21(29.6) | 0.49 |
| Lower respiratory infection (LRI) | 42(28.6) | 25(32.9) | 17(23.9) | 0.28 |
| Thrombocytopenia | 41(27.9) | 22(28.9) | 19(26.8) | 0.86 |
| Cough | 38(25.9) | 23(30.3) | 15(21.1) | 0.25 |
| Diarrhea | 30(20.4) | 19(25.0) | 11(15.5) | 0.21 |
| Chill | 25(17.0) | 16(21.1) | 9(12.7) | 0.19 |
| Scheming damage | 24(16.3) | 13(17.1) | 11(15.5) | 0.84 |
| Rash | 22(15.0) | 13(17.1) | 9(12.7) | 0.51 |
| Swirl | 22(15.0) | 10(13.2) | 12(16.9) | 0.66 |
| Chest distress | 22(15.0) | 14(18.4) | 8(11.3) | 0.25 |
| Expectoration | 20(13.6) | 12(15.8) | 8(11.3) | 0.47 |
| Palpitation | 19(12.9) | 6(7.9) | 13(18.3) | 0.08 |
| Palpebral conjunctival hyperemia | 18(12.2) | 10(13.2) | 8(11.3) | 0.81 |
| Throat pain | 16(10.9) | 10(13.2) | 6(8.5) | 0.42 |
| Anhelation | 15(10.2) | 9(11.8) | 6(8.5) | 0.58 |
| Skin itch | 14(9.5) | 7(9.2) | 7(9.9) | 1.00 |
| Flustered | 13(8.8) | 6(7.9) | 7(9.9) | 0.75 |
| Emesis | 12(8.2) | 4(5.3) | 8(11.3) | 0.22 |
| Pleurisy | 11(7.5) | 6(7.9) | 5(7.0) | 1.00 |
| Acute renal insufficiency | 11(7.5) | 7(9.2) | 4(5.6) | 0.55 |
| Stomachache | 10(6.8) | 4(5.3) | 6(8.5) | 0.53 |
| Hyperlipidaemia | 11(7.5) | 6(7.9) | 5(7.0) | 1.00 |
| Hyperuricemia | 22(15.0) | 17(22.4) | 5(7.0) | 0.01 |
| Hypohepatia | 14(9.5) | 10(13.2) | 4(5.6) | 0.16 |
| Electrolyte metabolism disorder | 15(10.2) | 8(10.5) | 7(9.9) | 1.00 |
| Hypokalemia | 46(31.3) | 17(22.4) | 29(40.8) | 0.02 |
| Hyponatremia | 6(4.1) | 5(6.6) | 1(1.4) | 0.22 |
| Hypoproteinemia | 25(17.0) | 12(15.8) | 13(18.3) | 0.83 |
| Mild anemia | 13(8.8) | 5(6.6) | 8(11.3) | 0.38 |
| Moderate anemia | 8(5.4) | 4(5.3) | 4(5.6) | 1.00 |
| Pharyngohemia | 7(4.8) | 5(6.6) | 2(2.8) | 0.45 |
| Gastrointestinal dysfunction | 7(4.8) | 3(3.9) | 4(5.6) | 0.72 |
| Antiadoncus | 5(3.4) | 4(5.3) | 1(1.4) | 0.36 |
| Rickettsial infection | 4(2.7) | 4(5.3) | 0(0.0) | 0.11 |
| Upper respiratory infection (URI) | 3(2.0) | 1(1.3) | 2(2.8) | 0.61 |
| Orbital pain | 2(1.4) | 0(0.0) | 2(2.8) | 0.24 |
| Breathing difficulties | 2(1.4) | 0(0.0) | 2(2.8) | 0.23 |
| Epistaxis | 4(2.7) | 2(2.6) | 2(2.8) | 1.00 |
| Bleeding gums | 2(1.4) | 1(1.3) | 1(1.4) | 1.00 |
| Face red | 2(1.4) | 2(2.6) | 0(0.0) | 0.53 |
| Acute Respiratory Distress Syndrome | 2(1.4) | 2(2.6) | 0(0.0) | 0.51 |

Table D. The univariate and multivariate analysis of severe dengue infection.

| **Characteristic** | **Univariate Analysis**  **OR (95%CI)** | **Multivariable Analysis**  **OR (95%CI)** |
| --- | --- | --- |
| Age group |  |  |
| ≤10 | 1.00 |  |
| 10~20 | - |  |
| 20~30 | 2.00(0.16-48.32) |  |
| 30~40 | 1.25(0.17-25.71) |  |
| 40~50 | 1.14(0.14-24.04) |  |
| 50~60 | 0.50(0.02-13.76) |  |
| 60~70 | 2.00(0.24-42.62) |  |
| 70~ | 0.73(0.03-20.28) |  |
| Ethnicity |  |  |
| Han | 1.00 |  |
| Dai | 0.65(0.10-2.58) |  |
| Other | 1.14(0.17-4.80) |  |
| Geographic location |  |  |
| Gengma | 1.00 |  |
| Other counties | 1.27(0.38-3.67) |  |
| Symptoms |  |  |
| Fever | 0.83(0.13-16.16) |  |
| Weakness | 1.47(0.38-9.73) |  |
| Poor appetite | 3.22(0.86-20.99) |  |
| Stiff | 0.74(0.27-2.28) |  |
| Chilly | 0.96(0.35-2.77) |  |
| Leukopenia | 0.90(0.33-2.44) |  |
| Nausea | 2.11(0.78-6.08) |  |
| Headache | 1.26(0.46-3.44) |  |
| Liver damage | 2.07(0.76-5.68) |  |
| Hypokalemia | 0.83(0.25-2.35) |  |
| Lower respiratory infection (LRI) | 2.91(1.05-8.07) * |  |
| Thrombocytopenia | 3.03(1.10-8.42) * | 3.06(1.05-9.03) * |
| Cough | 2.01(0.69-5.57) |  |
| Cyst of kidney | 1.08(0.29-3.30) |  |
| Diarrhea | 2.19(0.70-6.25) |  |
| Chill | 1.47(0.39-4.58) |  |
| Hypoproteinemia | 2.10(0.62-6.26) |  |
| Scheming damage | 3.08(0.97-9.06) |  |
| Rash | 1.16(0.25-3.94) |  |
| Swirl | 0.30(0.02-1.60) |  |
| Chest distress | 1.16(0.25-3.94) |  |
| Expectoration | 2.02(0.52-6.48) |  |
| Palpitation | 0.82(0.12-3.25) |  |
| Palpebral conjunctival hyperemia | 3.43(0.98-10.83) |  |
| Anhelation | 1.12(0.16-4.55) |  |
| Electrolyte metabolism disorder | 3.06(0.77-10.41) |  |
| Skin itch | 0.52(0.03-2.90) |  |
| Hypohepatia | 5.13(1.40-17.36) ** | 3.82(1.00-13.61) * |
| Mild anemia | 1.34(0.20-5.61) |  |
| Flustered | 1.34(0.20-5.61) |  |
| Emesis | 2.67(0.55-10.14) |  |
| Type 2 diabetes | 1.49(0.22-6.32) |  |
| Hyperlipidaemia | 1.67(0.24-7.22) |  |
| Pleurisy | 1.67(0.24-7.22) |  |
| Acute renal insufficiency | 0.70(0.04-4.01) |  |
| Stomachache | 1.89(0.27-8.40) |  |
| Splenauxe | 3.49(0.70-14.09) |  |
| Moderate anemia | 2.56(0.36-12.27) |  |
| Hypocalcemia | 1.02(0.05-6.28) |  |
| Secondary hypertension | 1.03(0.05-6.28) |  |
| Hyponatremia | 1.46(0.07-9.79) |  |
| Lacunar infarction | 1.46(0.07-9.79) |  |
| Breathing difficulties | 7.53(0.29-196.42) |  |
| Bleeding gums | 7.53(0.29-196.42) |  |
| Face red | 7.53(0.29-196.42) |  |
| Primary hypertension | 7.53(0.29-196.42) |  |

*p<0.05, **p<0.01, ***p<0.001.
